# Integrated Prenatal Genetic Evaluation of Renal Agenesis: Chromosomal Microarray Analysis, Whole Exome Sequencing, and Outcome Correlations in 203 Fetuses

**DOI:** 10.3390/genes17020176

**Published:** 2026-01-31

**Authors:** Na Zhang, Ruibin Huang, Fang Fu, Hang Zhou, Ru Li, Can Liao

**Affiliations:** 1 The First Affiliated Hospital of Jinan University, Jinan University, Guangzhou 510620, China; nzhang2021@163.com; 2 Prenatal Diagnostic Center, Guangzhou Women and Children’s Medical Center, Guangzhou Medical University, Guangzhou 510620, China; huangruibin96@hotmail.com (R.H.); fuyingyi2008@163.com (F.F.); gmuzhouhang@163.com (H.Z.); linra020@126.com (R.L.)

**Keywords:** renal agenesis, chromosomal microarray analysis, whole exome sequencing, prenatal diagnosis

## Abstract

Objectives: To characterize the prenatal phenotypic spectrum, genetic findings, and pregnancy outcomes of fetal renal agenesis (RA), and to clarify the complementary roles of chromosomal microarray analysis (CMA) and whole exome sequencing (WES) in phenotype-stratified prenatal evaluation. Methods: This retrospective study included 203 RA fetuses between March 2017 and November 2025. All cases underwent genome-wide copy number variant (CNV) analysis, and selected cases underwent WES. Detection rates were compared across subgroups by laterality, isolated vs. non-isolated phenotype, fetal sex, and presence of extrarenal anomalies. Pregnancy outcomes and postnatal imaging follow-up were collected when available. A systematic literature review of prenatal genetic testing in RA fetuses was performed. Results: Among 203 fetuses, unilateral RA accounted for 92.6% of cases, and 65.0% were isolated. Chromosomal abnormalities were identified in 15 fetuses (7.4%), including aneuploidies and pathogenic or likely pathogenic (P/LP) CNVs. WES identified P/LP single nucleotide variants in 8 of 127 cases (6.3%), increasing to 8.7% when variants with potential clinical relevance were included. Diagnostic yield of WES was significantly higher in bilateral RA, non-isolated cases, and fetuses with extrarenal anomalies. Postnatal follow-up confirmed RA in most liveborn cases, although additional phenotypes emerged in some children. Literature synthesis identified recurrent CNVs at 16p11.2 and 22q11.21 and frequent involvement of *FRAS1*, *FREM2*, *GFRA1*, and *GREB1L*. Conclusions: RA shows marked phenotypic and genetic heterogeneity. CMA remains a first-tier prenatal test, while WES provides substantial incremental yield in bilateral, non-isolated, or extrarenal-associated RA. Integrated, phenotype-driven testing with longitudinal follow-up supports improved prognostication and genetic counseling.

## 1. Introduction

Renal agenesis (RA) is a severe developmental anomaly within the spectrum of congenital anomalies of the kidney and urinary tract (CAKUT), characterized by the complete absence of one or both kidneys due to disrupted nephrogenesis [1]. Population-based studies estimate the incidence of unilateral renal agenesis (URA) at approximately 1 in 1000–2000 births, whereas bilateral renal agenesis (BRA) is much rarer, occurring in approximately 1 in 3000–10,000 births [1,2,3]. The clinical consequences of RA are strongly dependent on laterality: URA is often compatible with long-term survival, while BRA is typically lethal because of severe oligohydramnios, pulmonary hypoplasia, and Potter sequence [1]. With the widespread use of second-trimester prenatal ultrasonography, RA is increasingly detected in utero, frequently in association with reduced amniotic fluid volume or additional structural anomalies [4]. Nevertheless, prenatal counseling remains particularly challenging, as fetal imaging alone often fails to reliably predict postnatal outcome, recurrence risk, and long-term prognosis.

Genetic heterogeneity represents a major contributor to the clinical complexity of RA. Both chromosomal abnormalities and monogenic disorders have been implicated in unilateral and bilateral forms [5,6,7,8,9,10,11,12,13,14]. Pathogenic copy number variants (CNVs), particularly recurrent microdeletions and microduplications involving 17p12 [11,12], 22q11.2 [5,6,9,12,15], and 16p11.2 [5,9,12], account for a proportion of cases, often through dosage-sensitive renal developmental genes such as *HNF1B* [16,17] and *LHX1* [18]. Consequently, chromosomal microarray analysis (CMA) has become a first-tier genetic test for fetuses with CAKUT [19]. However, several studies have demonstrated that the diagnostic yield of CMA in isolated URA is relatively low, whereas its contribution is more substantial in non-isolated cases [5,6,12]. In contrast, whole exome sequencing (WES), particularly trio-based approaches, has emerged as a powerful tool for identifying monogenic causes of RA, especially in BRA, RA with extrarenal anomalies, or recurrent cases [7,8,10,13,14], revealing pathogenic variants in genes such as *GREB1L* [20,21,22], *FRAS1* [10,14], *FREM2* [14,23], *NPNT* [24], and *GFRA1* [13,25]. Despite these advances, the optimal integration of CMA and WES in prenatal RA evaluation remains debated.

To date, most published studies have focused on selected subgroups of RA or applied heterogeneous testing strategies, limiting the direct comparison of diagnostic yields and their implications for prenatal counseling. In particular, data integrating detailed prenatal imaging, comprehensive genetic testing, and pregnancy outcomes within a single cohort remain scarce. In the present study, we retrospectively analyzed 203 RA fetuses at our center, systematically summarizing their prenatal imaging features, genetic testing results, and pregnancy outcomes. In parallel, we integrated evidence from previously published studies to evaluate the diagnostic performance of CMA and next-generation sequencing (NGS)-based testing across different RA subtypes. By combining institutional data with the existing literature, we aim to clarify the respective roles of CMA and WES in the prenatal evaluation of renal agenesis and to provide an evidence-based framework for prognostication and genetic counseling.

## 2. Materials and Methods

### 2.1. Study Design and Cohort

This retrospective study included 203 RA fetuses who were referred to the Prenatal Diagnosis Center of Guangzhou Women and Children’s Medical Center (GWCMC) between March 2017 and November 2025. The study protocol was approved by the Ethics Committee of GWCMC, and written informed consent was obtained from all participants. This retrospective cohort was conducted as part of the Specialist Alliance for Prevention and Control of Birth Defects (SAPCBD), which was initiated in 2021 by the Prenatal Diagnostic Center of GWCMC and consisted of 66 institutions in more than 10 provinces and has facilitated prenatal diagnostic evaluation in nearly 30,000 fetuses to date.

Clinical data were retrospectively collected from electronic medical records and included maternal and paternal age, gestational age at diagnosis, fetal sex, prenatal imaging findings, genetic testing results, and available pregnancy outcome information. Based on prenatal ultrasonographic findings, cases were classified as isolated or non-isolated RA. Isolated RA was defined as the presence of renal agenesis without additional congenital anomalies, whereas non-isolated RA was defined as renal agenesis accompanied by other anomalies, including additional CAKUT, soft markers, or extrarenal structural anomalies.

### 2.2. Genetic Testing and Variant Interpretation

Depending on gestational age and clinical indications, fetal samples were obtained from amniotic fluid, cord blood, or products of conception, while parental samples were collected from peripheral blood. Genomic DNA was extracted using standard protocols according to the manufacturer’s instructions after written informed consent was obtained.

All fetal samples initially underwent quantitative fluorescent polymerase chain reaction (QF-PCR) to exclude maternal cell contamination and to rapidly screen for common aneuploidies involving chromosomes 13, 18, 21, X, and Y. Genome-wide CNV analysis was subsequently performed using CMA in most cases, while CNV sequencing (CNV-seq) was applied in a small number of cases (*n* = 9). CMA was performed using the Affymetrix CytoScan HD or CytoScan 750K arrays (Affymetrix, Santa Clara, CA, USA), which integrate single-nucleotide polymorphism (SNP) array and array-based comparative genomic hybridization (aCGH) technologies, with an effective resolution of approximately 10 kb for deletions and 100 kb for duplications. CNV-seq was conducted using low-coverage whole genome sequencing to enable genome-wide detection of copy-number changes. All CNVs identified by either method were interpreted using the same clinical criteria and annotated according to the GRCh37/hg19 reference genome.

Trio-WES was performed in selected cases based on predefined clinical indications, including CNV analysis results that were negative or classified as variants of uncertain significance (VUS), sufficient DNA quantity and quality, and availability of parental samples. In addition, in some cases with advanced gestational age or strong parental preference, trio-WES was performed concurrently with CNV analysis as part of a one-step prenatal diagnostic approach. Targeted exome capture was carried out using the Agilent SureSelect Human All Exon V6 kit (Agilent Technologies, Santa Clara, CA, USA), followed by paired-end sequencing (150 bp reads) on Illumina HiSeq 2500, HiSeq X Ten, or NovaSeq platforms (Illumina, San Diego, CA, USA). Bioinformatic analysis of exome sequencing data was performed using an in-house pipeline, including sequence alignment, variant calling, quality control, variant filtration, and annotation. Variant interpretation incorporated inheritance patterns, fetal phenotypes, and family segregation analysis, with confirmation of candidate variants by Sanger sequencing when applicable. Sequence variants were classified according to the American College of Medical Genetics and Genomics (ACMG) and Association for Molecular Pathology (AMP) guidelines as pathogenic (P), likely pathogenic (LP), VUS, likely benign, or benign. For the purpose of diagnostic yield calculation, only P and LP variants were considered positive findings. Incidental findings (IFs) were reported in accordance with current ACMG recommendations. Prior to trio-WES, all families received pre-test genetic counseling, and written informed consent explicitly addressed the possibility of detecting incidental findings unrelated to the fetal renal phenotype, as well as the institutional reporting policy for such results. Detailed information regarding the analysis and interpretation of the WES data is provided in Supplementary File S1.

### 2.3. Statistical Analysis

Statistical analyses were performed using SPSS software (version 26.0; IBM Corp., Armonk, NY, USA). Categorical variables, including detection rates across clinical subgroups stratified by laterality (URA vs. BRA), fetal sex, isolated versus non-isolated renal agenesis, and the presence or absence of extrarenal anomalies, were summarized as counts and percentages and compared using Fisher’s exact test. Continuous variables, including maternal age, paternal age, and gestational age at diagnosis, were expressed as mean ± standard deviation (SD) and compared between groups using Welch’s *t*-test. All statistical tests were two-sided, and *p*-value < 0.05 was considered statistically significant.

## 3. Results

### 3.1. Clinical Characteristics and Prenatal Imaging Features of RA Fetuses

A total of 203 fetuses diagnosed with RA were included in this study. All cases were referred to the Prenatal Diagnosis Center of GWCMC between March 2017 and November 2025. The mean maternal age at diagnosis was 30.14 ± 4.06 years, and the mean paternal age was 32.34 ± 4.98 years. The mean gestational age at diagnosis was 24.72 ± 3.18 weeks. Among the affected fetuses, 77 (37.9%, 77/203) were male and 126 (62.1%, 126/203) were female. Most cases were diagnosed during the second trimester and underwent amniocentesis, accounting for 172 fetuses (84.7%, 172/203). Twenty-two cases (10.8%, 22/203) were diagnosed at a later gestational age and underwent percutaneous umbilical blood sampling. The remaining nine cases (4.4%, 9/203) were diagnosed in the context of severe oligohydramnios or anhydramnios and underwent genetic testing using products of conception. Notably, all nine cases with pregnancy tissue samples showed bilateral renal involvement. RA predominantly presented as unilateral involvement, accounting for 188 cases (92.6%, 188/203), whereas bilateral renal agenesis was observed in 15 cases (7.4%, 15/203). Among unilateral cases, left-sided RA was identified in 86 (42.4%, 86/203) fetuses and right-sided RA in 102 (50.2%, 102/203) fetuses. When RA and concomitant CAKUT were considered together, renal involvement was unilateral in 172 (84.7%, 172/203) fetuses and bilateral in 31 (15.3%, 31/203) fetuses.

Based on prenatal ultrasonographic findings, 132 (65.0%, 132/203) cases were classified as isolated, while 71 (35.0%, 71/203) cases were classified as non-isolated, showing additional CAKUT features, soft markers, or extrarenal structural anomalies. A total of 17 (8.4%, 17/203) fetuses exhibited additional CAKUT phenotypes, including contralateral duplicated kidney or duplex collecting system (*n* = 5), compensatory enlargement of the contralateral kidney (*n* = 2), contralateral renal dysplasia (*n* = 5), contralateral hydronephrosis or pyelectasis (*n* = 3), renal cysts (*n* = 1), and ectopic kidney (*n* = 1). Extrarenal structural anomalies were identified in 46 (22.7%, 46/203) fetuses. Cardiovascular anomalies were the most frequent, affecting 14 cases, including ventricular septal defects in 8 fetuses. Other extrarenal anomalies included multisystem anomalies (*n* = 10), skeletal anomalies (n = 8), fetal growth restriction (*n* = 8), central nervous system anomalies (*n* = 3), gastrointestinal anomalies (*n* = 2), and respiratory system anomalies (*n* = 1). Soft markers were detected in 36 (17.7%, 36/203) fetuses, with single umbilical artery being the most common finding, present in 20 cases. Oligohydramnios or anhydramnios was observed in 20 (9.9%, 20/203) fetuses, including 15 BRA cases, four URA cases combined with contralateral renal abnormalities, and one case with isolated left RA associated with multiple extrarenal anomalies.

### 3.2. Diagnostic Yield and Spectrum of Chromosomal Abnormalities

CMA was performed in all 203 fetuses with renal agenesis. Clinically significant chromosomal abnormalities were identified in 15 cases, yielding an overall diagnostic rate of 7.4% (15/203). VUS were detected in four additional cases (2.0%, 4/203). Among the 15 fetuses with clinically significant chromosomal abnormalities detected by CMA, four cases were diagnosed with whole-chromosome aneuploidies, including trisomy 18 (*n* = 1), 47, XXY (*n* = 1), 47, XXX (*n* = 1), and mosaic 47, XXX (*n* = 1). The remaining 11 cases harbored P/LP CNVs beyond whole-chromosome aneuploidies, including nine deletions and two duplications. Six of the 15 fetuses presented with isolated RA, whereas the remaining nine cases were classified as non-isolated RA and were associated with additional CAKUT features, extrarenal anomalies, or both. Parental studies were available for nine cases, confirming six de novo abnormalities and three inherited abnormalities; inheritance could not be determined in the remaining cases. Detailed genomic coordinates, CNV sizes, inheritance patterns, associated phenotypes, and pregnancy outcomes are summarized in Table 1.

Stratified analyses according to laterality, fetal sex, the presence of associated anomalies, and the presence of extrarenal anomalies revealed no statistically significant differences in the detection rate of clinically significant chromosomal abnormalities. Specifically, the detection rates were 7.4% (14/188) in URA and 6.7% (1/15) in BRA cases (*p* = 1.000), and 10.4% (8/77) in male fetuses versus 5.6% (7/126) in female fetuses (*p* = 0.269). The detection rate was 5.3% (7/132) in isolated and 11.3% (8/71) in non-isolated cases (*p* = 0.159), and was higher in fetuses with extrarenal anomalies compared with those without extrarenal anomalies [11.1% (7/63) vs. 5.7% (8/140)], although the difference was not statistically significant (*p* = 0.244). Comparisons of continuous clinical variables showed no significant differences between CMA-positive and CMA-negative groups, including maternal age (30.2 ± 3.3 vs. 30.1 ± 4.1 years, *p* = 0.913), paternal age (31.5 ± 4.1 vs. 32.4 ± 4.9 years, *p* = 0.431), or gestational age at diagnosis (25.7 ± 3.7 vs. 24.6 ± 3.1 weeks, *p* = 0.279).

### 3.3. Diagnostic Yield and Molecular Spectrum of Trio-WES

Trio-WES was subsequently performed in 127 RA fetuses, and eight P/LP single nucleotide variants (SNVs) were identified, yielding an overall diagnostic rate of 6.3% (8/127). When fetuses carrying LP/VUS compound heterozygous variants were additionally included, a total of 11 fetuses (8.7%, 11/127) were found to harbor SNVs with potential clinical relevance (defined as P/LP and LP/VUS variants). In addition to these findings, VUS were identified in eight fetuses (6.3%, 8/127). IFs unrelated to the fetal renal phenotype were detected in three cases (2.4%, 3/127), including two fetuses with pathogenic *GJB2* variants associated with hearing loss and one fetus with a pathogenic *PKP2* variant associated with arrhythmogenic cardiomyopathy (Table 2). Stratified analyses revealed significant differences in detection rates across clinical subgroups. The detection rate was significantly higher in BRA fetuses than in those with URA [30.8% (4/13) vs. 6.1% (7/114), *p* = 0.015]. Similarly, non-isolated was associated with a higher detection rate compared with isolated cases [15.8% (9/57) vs. 2.9% (2/70), *p* = 0.012], and fetuses with extrarenal anomalies showed a markedly higher yield than those without such abnormalities [18.4% (9/49) vs. 2.6% (2/78), *p* = 0.003]. In contrast, no significant difference in detection rate was observed between male and female fetuses [4.4% (2/45) vs. 11.0% (9/82), *p* = 0.326]. Comparisons of continuous variables showed no statistically significant differences in maternal age (29.2 ± 3.6 vs. 29.8 ± 4.0 years, *p* = 0.610) or paternal age (32.5 ± 3.4 vs. 32.1 ± 4.9 years, *p* = 0.727) between SNV-positive and SNV-negative cases. The gestational age at diagnosis tended to be lower in SNV-positive cases than in SNV-negative cases (22.5 ± 3.1 vs. 24.5 ± 3.3 weeks), although this difference did not reach statistical significance (*p* = 0.064).

Among the 11 fetuses harboring SNVs with potential clinical relevance, three carried compound heterozygous variants in *FRAS1*, which was the only gene identified in more than one case, whereas the remaining eight fetuses each harbored variants in distinct disease-associated genes, including *EYA1*, *KMT2A*, *FGFR2*, *PBX1*, *PUF60*, *KMT2D*, *FANCI*, and *LTBP3*. With respect to inheritance patterns, six cases were consistent with autosomal dominant inheritance, including five de novo variants and one variant inherited from the father, while the remaining five cases followed an autosomal recessive pattern, all of which carried compound heterozygous variants inherited from both parents. A total of 13 P/LP SNVs were identified in these cases. Variant classification revealed a predominance of loss-of-function variants, including five nonsense variants, three frameshift variants, and three canonical splice-site variants, accounting for the majority of pathogenic findings. In addition, one missense variant and one in-frame deletion were identified.

### 3.4. Pregnancy Outcomes and Postnatal Follow-Up

Among the 203 RA fetuses, pregnancy outcomes were successfully obtained for 149 cases, while 56 cases were lost to follow-up or lacked postnatal reassessment. A total of 59 pregnancies were terminated. Among these, 22 fetuses carried P/LP CNVs or SNVs, 18 were associated with additional extrarenal structural anomalies, six presented with BRA, two had URA combined with contralateral renal abnormalities and oligohydramnios, and one fetus carried a VUS SNV. The remaining ten pregnancies were terminated for other reasons. Eighty-eight fetuses were delivered and subsequently underwent postnatal imaging follow-up. Postnatal imaging confirmed the diagnosis of RA in 84 cases, among which compensatory hypertrophy of the contralateral kidney was observed in nine cases. In four cases, pelvic ectopic kidneys were diagnosed postnatally; none of these fetuses had undergone prenatal fetal magnetic resonance imaging (MRI). During postnatal follow-up, seven children were found to have newly identified clinical phenotypes that had not been detected prenatally, including scoliosis, sacrococcygeal teratoma, psychomotor developmental delay, ventricular septal defect, subependymal hemorrhage, cleft palate, and intracranial hemorrhage. In five of these cases, trio-WES had been performed prenatally with negative results, and reanalysis of the sequencing data after incorporating newly identified postnatal phenotypes remained negative.

### 3.5. Summary of Published Evidence on Prenatal Genetic Testing in RA

**Figure 1:**
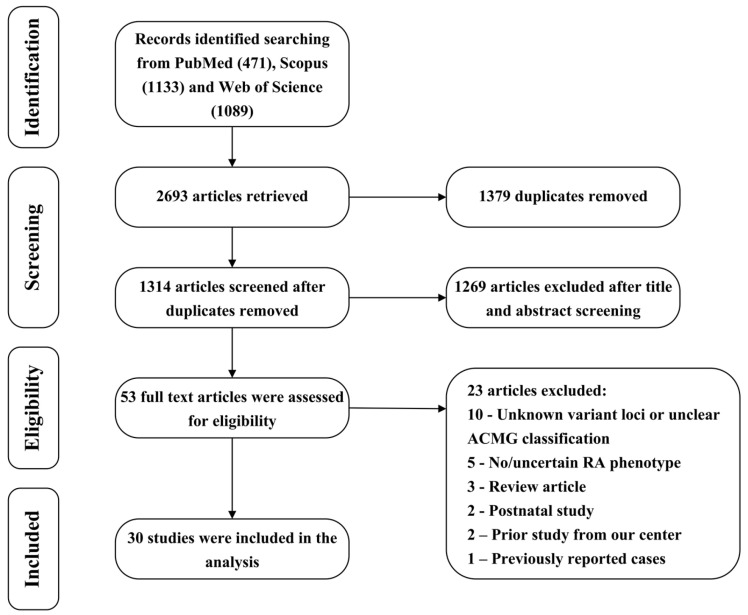
PRISMA flow diagram.

**Figure 2:**
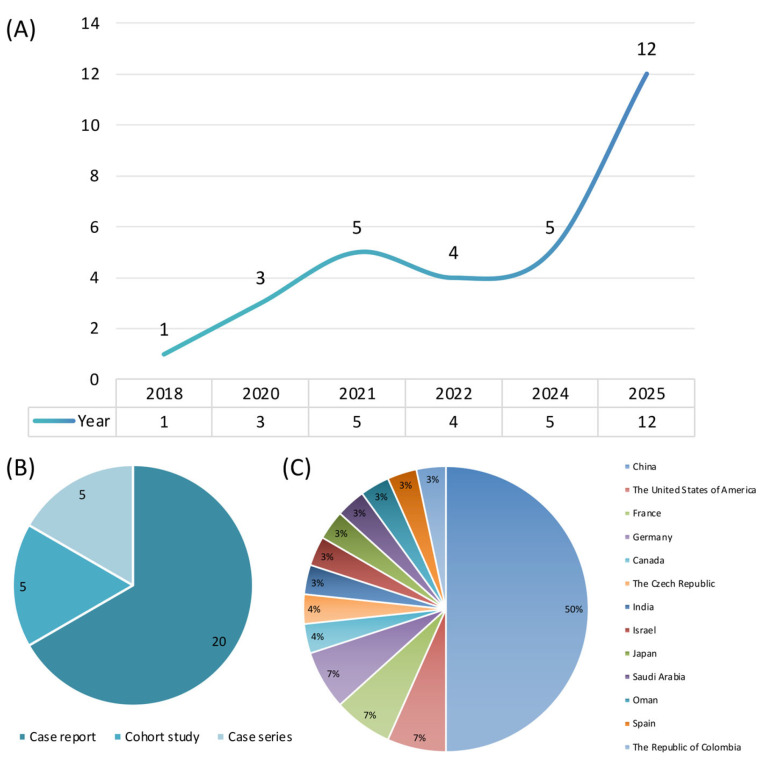
Trends in fetal renal agenesis research across publication years, study types, and countries. (**A**) Number of publications across years. (**B**) Distribution of study types. (**C**) Distribution of publications by country.

A systematic literature search and review identified a total of 30 studies reporting prenatal genetic testing in RA fetuses that were included for further analysis (Figure 1, Supplementary File S2). The included publications comprised five cohort studies [5,6,7,10,12], five case series [8,9,11,13,14], and twenty case reports [15,20,21,22,23,24,25,26,27,28,29,30,31,32,33,34,35,36,37,38]. With respect to publication year, one study was published in 2018, three in 2020, five in 2021, four in 2022, five in 2024, and twelve in 2025 (Figure 2). Geographically, the studies originated predominantly from China (*n* = 15), followed by the United States (*n* = 2), France (*n* = 2), Germany (*n* = 2), Canada (*n* = 1), the Czech Republic (*n* = 1), India (*n* = 1), Israel (*n* = 1), Japan (*n* = 1), Saudi Arabia (*n* = 1), Oman (*n* = 1), Spain (*n* = 1), and the Republic of Colombia (*n* = 1). Regarding genetic testing strategies, CMA was applied in six studies [5,6,9,11,12,15], targeted gene panels in two studies [10,23], genome sequencing (GS) in two studies [13,29], and WES in twenty studies [7,8,14,20,21,22,24,25,26,27,28,30,31,32,33,34,35,36,37,38]. Among the five cohort studies, three employed CMA and reported diagnostic yields ranging from 2.47% to 17.19%, with a pooled yield of 10.34% (24/232). One cohort study using a targeted gene panel reported a diagnostic yield of 15.38% (6/39), and one cohort study applying WES reported a diagnostic yield of 16.67% (2/12).

Across the 30 included studies, a total of 71 RA fetuses were reported to harbor P/LP genetic findings. Of these, 32 cases were identified by CMA, with the most frequently reported chromosomal abnormalities being 16p11.2 microdeletion (*n* = 7) and 22q11.21 microdeletion (*n* = 7). The remaining 39 cases were detected by NGS, most commonly involving variants in *FRAS1* (*n* = 8), followed by *FREM2* (*n* = 3), *GFRA1* (*n* = 3), *GREB1L* (*n* = 3), *PAX2* (*n* = 2), and *SALL4* (*n* = 2). Among the 71 genetically positive fetuses with RA reported in the literature, 35 presented with isolated renal agenesis. Of these, 19 cases (54.3%) were attributed to CNVs. Recurrent CNVs predominantly involved 22q11.21 microdeletion (5/35, 14.3%) and 16p11.2 copy-number alterations (5/35, 14.3%), including four microdeletions and one microduplication, followed by 17p12 microdeletion (2/35, 5.7%), whereas all other CNVs were reported only once. The remaining 16 cases (45.7%) harbored sequence-level variants identified by NGS, with the most frequently implicated genes being *GFRA1* (3/35, 8.6%) and *GREB1L* (3/35, 8.6%), followed by *FRAS1* (2/35, 5.7%) and *FREM2* (2/35, 5.7%); all other genes were reported in single cases.

## 4. Discussion

### 4.1. Principal Findings and Clinical Relevance

In this study, we systematically evaluated the prenatal phenotype, genetic findings, and pregnancy outcomes of a large cohort of 203 RA fetuses and further integrated evidence from previously published studies. Using a combined strategy of genome-wide CNV analysis and trio-WES, we demonstrated that clinically significant genetic abnormalities are identifiable in a substantial proportion of RA cases, with distinct diagnostic yields across different clinical subgroups. Chromosomal abnormalities accounted for 7.4% of cases, whereas trio-WES provided an additional diagnostic yield of 6.3% based on P/LP variants, increasing to 8.7% when variants with potential clinical relevance were considered. Notably, the diagnostic yield of WES was significantly enriched in BRA fetuses, non-isolated phenotypes, and extrarenal structural anomalies. These findings highlight the pronounced genetic heterogeneity underlying RA and underscore the clinical value of a phenotype-driven, stepwise genetic testing strategy for optimizing prenatal counseling and risk assessment.

### 4.2. Prenatal Phenotype Spectrum and Counseling Challenges

Renal agenesis exhibited marked phenotypic heterogeneity in our cohort, posing substantial challenges for prenatal counseling. Although unilateral renal agenesis predominated, more than one-third of fetuses presented with non-isolated phenotypes, including additional CAKUT features, soft markers, or extrarenal structural anomalies, most frequently involving the cardiovascular system. In addition, oligohydramnios or anhydramnios was almost exclusively observed in fetuses with BRA or URA accompanied by contralateral renal abnormalities, underscoring the close relationship between laterality, renal functional reserve, and amniotic fluid volume. These findings are consistent with previous reports indicating that the prenatal presentation of RA spans a broad clinical spectrum and that sonographic features alone are often insufficient to accurately predict postnatal outcome.

Importantly, our postnatal follow-up further highlighted the limitations of prenatal imaging. Pelvic ectopic kidneys were identified after birth in four fetuses initially diagnosed with RA, none of whom had undergone fetal MRI, suggesting that renal malposition may be misclassified as agenesis in the prenatal setting. Moreover, additional clinical phenotypes emerged postnatally in a subset of children, several of whom had undergone prenatal trio-WES with negative results and remained negative after reanalysis incorporating newly identified features. Together, these observations emphasize that prenatal assessment of RA is inherently constrained by incomplete phenotypic information and evolving postnatal manifestations, reinforcing the need for integrated genetic testing strategies and longitudinal follow-up to improve prognostic accuracy and counseling.

### 4.3. Chromosomal Abnormalities and the Role of CNV Analysis in Fetal RA

In our cohort, clinically significant chromosomal abnormalities were identified in 7.4% of fetuses with renal agenesis by genome-wide CNV analysis, supporting the role of CNV testing as a foundational component of prenatal genetic evaluation in RA. This diagnostic yield falls within the range reported by previous cohort studies, in which CMA-based detection rates varied from approximately 2% to 17%, with an overall yield of around 10% [5,6,12]. Consistent with prior reports, the majority of pathogenic CNVs identified in our study were deletions rather than duplications, and recurrent regions such as 16p11.2 and 22q11.21 were among the most frequently implicated loci in the published literature, underscoring their relevance to renal development. Despite these findings, CNV analysis demonstrated limited discriminatory power across clinical subgroups in our cohort. Although higher detection rates were observed in non-isolated cases and in fetuses with extrarenal anomalies, these differences did not reach statistical significance, likely reflecting the relatively small number of CNV-positive cases and the genetic heterogeneity of RA. These negative subgroup comparisons should be interpreted cautiously given limited statistical power. Therefore, larger multicenter cohorts and/or meta-analyses will be necessary to robustly evaluate subgroup-specific CMA yields and to determine whether clinically meaningful differences exist across phenotypic strata. Importantly, a subset of isolated RA cases also harbored pathogenic chromosomal abnormalities, indicating that the absence of additional sonographic anomalies does not preclude an underlying chromosomal etiology. Taken together, these observations suggest that CNV analysis should be routinely considered in the prenatal evaluation of RA, irrespective of phenotypic complexity, while acknowledging that its diagnostic yield alone may be insufficient to fully elucidate the genetic basis of the condition.

### 4.4. Added Value of Trio-WES and Gene-Disease Correlations in Fetal RA

Our study further confirms the incremental diagnostic value of trio-WES in the prenatal evaluation of RA, particularly when applied in a phenotype-stratified manner. Although the overall diagnostic yield of trio-WES based on P/LP variants was relatively modest (6.3%), the detection rate increased significantly in specific clinical subgroups, including fetuses with BRA, non-isolated RA, and those with extrarenal structural anomalies. In fetuses with BRA, the diagnostic yield approached one third, indicating that monogenic etiologies play a particularly prominent role in severe or syndromic forms of RA. These findings are highly consistent with previous reports, in which exome sequencing was preferentially applied to selected subgroups of fetuses with RA and yielded diagnostic rates typically ranging from 15% to 17% [7,10].

In our cohort, sequence-level variants in *KMT2A* and *FRAS1* were identified in fetuses with isolated RA. When integrated with previously published studies, isolated RA has been associated with a diverse set of disease-associated genes, including *KMT2A*, *FRAS1* [14], *FREM1* [37], *FREM2* [14], *GFRA1* [13,25], *GREB1L* [20,21,22], *PAX2* [28], *NPNT* [24], *EYA1* [10], *HOXC4* [8], and *PRPF8* [8] (Table 3). Although each of these genes has been reported in only a limited number of cases, they converge on key biological pathways essential for kidney development, supporting the presence of genuine monogenic etiologies even in apparently isolated presentations. These genes play critical roles in early renal and urinary tract development, including ureteric bud induction, metanephric mesenchyme differentiation, extracellular matrix homeostasis, and epithelial–mesenchymal interactions. Disruption of these pathways may impair ureteric bud formation or reciprocal signaling, ultimately resulting in renal developmental failure or complete agenesis.

Of particular note, *FRAS1*, *FREM1* and *FREM2* encode components of the Fraser protein complex, which is essential for maintaining epithelial–mesenchymal structural integrity during embryogenesis [39]. Pathogenic variants in these genes cause Fraser syndrome, a multisystem developmental disorder characterized in both humans and animal models by cryptophthalmos, syndactyly, and genitourinary anomalies, among which RA represents a common and clinically important feature [40]. Experimental studies in animal models have demonstrated that loss of function of the Fraser complex disrupts basement membrane integrity and interferes with organ morphogenesis, providing a clear mechanistic explanation for renal and urinary tract involvement [41,42,43]. The identification of variants in these genes in RA fetuses in our study, even in the absence of overt syndromic features at the time of prenatal assessment, suggests that RA may represent an early or incomplete manifestation within a broader spectrum of developmental disorders.

Taken together, these findings indicate that isolated RA should not be regarded as a genetically “low-risk” phenotype. Instead, selective application of WES may be justified in carefully phenotyped isolated cases, particularly when combined with detailed prenatal assessment and longitudinal postnatal follow-up.

### 4.5. Pregnancy Outcomes and Implications for Prenatal Counseling

Pregnancy outcomes in our cohort further underscore the importance of integrated prenatal assessment in the management of RA fetuses. Termination of pregnancy was more frequently observed in cases with severe phenotypes, including BRA, oligohydramnios or anhydramnios, the presence of extrarenal structural anomalies, and the identification of P/LP genetic variants. This pattern is consistent with current clinical practice, in which the severity of fetal structural abnormalities and their potential genetic etiology substantially influence parental decision-making.

Among fetuses that were delivered and successfully followed postnatally, the diagnosis of RA was confirmed in most cases, and compensatory hypertrophy of the contralateral kidney was observed in a subset of children with URA, indicating favorable physiological adaptation. It should be noted that a proportion of cases were lost to follow-up or lacked postnatal reassessment, which is common in real-world practice at tertiary referral centers, as many families deliver and continue pediatric care at local hospitals, relocate, or are unable/unwilling to return for follow-up visits. This incomplete follow-up may introduce bias in outcome estimation and could lead to under-recognition of later-onset or milder postnatal phenotypes. However, postnatal follow-up also revealed newly identified clinical phenotypes that had not been detected prenatally, including neurodevelopmental abnormalities, congenital heart defects, and anomalies involving other organ systems. Notably, in several of these cases, prenatal trio-WES had yielded negative results, and reanalysis of the sequencing data after incorporating newly identified postnatal phenotypes remained negative. This may reflect both technical and interpretative limitations of exome-based testing, including reduced sensitivity for non-coding or deep intronic variants, low-level mosaicism, and certain structural rearrangements; moreover, even with updated phenotypic information, the available evidence supporting some gene-disease associations or variant-level pathogenicity may remain insufficient to enable definitive classification, particularly for rare or novel variants. In addition, incomplete penetrance, variable expressivity, and potential oligogenic/multifactorial mechanisms may further increase the complexity of genotype–phenotype matching. These findings highlight the current technical and interpretative limitations of prenatal genetic testing and reflect the dynamic and evolving nature of genotype–phenotype correlations.

Beyond the diagnostic yield for RA, trio-WES may also reveal IFs unrelated to the fetal renal phenotype but potentially relevant to postnatal care. In our cohort, IFs were identified in 2.4% (3/127) of RA fetuses, including two cases with pathogenic homozygous *GJB2* variants associated with hearing loss and one case with a pathogenic heterozygous *PKP2* variant associated with arrhythmogenic cardiomyopathy. Although these results did not explain RA, they may still inform postnatal management, such as early audiological evaluation and timely cardiology assessment when appropriate. Therefore, pre-test counseling and written informed consent should explicitly address the possibility of non-kidney diagnoses and clarify the institutional reporting policy for such findings, together with plans for post-test genetic counseling and follow-up.

From a clinical management perspective, our findings support a tiered, phenotype-driven genetic testing strategy for RA fetuses. CMA should remain the first-tier test for the detection of chromosomal abnormalities, whereas trio-WES can substantially increase the diagnostic yield in selected cases, particularly those with BRA, non-isolated phenotypes, or associated extrarenal structural anomalies. However, in time-sensitive prenatal diagnostic settings, a strictly sequential workflow may be constrained by gestational age and test turnaround time; it is generally feasible in early pregnancy and the early second trimester. For late-presenting cases-especially those beyond 25 weeks of gestation-we recommend a “one-step” approach, performing CNV analysis and trio-WES concurrently, to shorten time-to-results and support timely decision-making. At the same time, the persistence of unresolved cases despite the emergence of additional postnatal phenotypes underscores the importance of long-term follow-up and periodic data reanalysis, rather than the premature exclusion of a genetic etiology. Accordingly, prenatal counseling for renal agenesis should not rely solely on isolated imaging or genetic findings but should integrate structural severity, genetic testing results, and postnatal follow-up information. Such an approach enables more refined risk stratification, avoids overly deterministic prognostic conclusions, and provides families with more comprehensive and individualized decision-making support.

### 4.6. Strengths and Limitations

The major strengths of this study include the large cohort from a tertiary referral center with a standardized clinical workflow, comprehensive genetic testing, and pregnancy outcome data, and the parallel synthesis of institutional findings with published evidence. The single-center design enabled consistent phenotyping, uniform laboratory procedures, and harmonized variant interpretation across the entire cohort. By applying both CMA and trio-WES in a phenotype-stratified manner, we were able to directly compare their diagnostic performance across different subtypes of RA and to delineate clinical scenarios in which each modality provides the greatest utility. Importantly, the inclusion of postnatal follow-up and reanalysis further enhances the clinical relevance of our findings and reflects real-world prenatal diagnostic practice.

Several limitations should be acknowledged. First, this was a retrospective study conducted at a single tertiary referral center, which may introduce referral bias toward more complex or severe phenotypes, and may limit the generalizability of our diagnostic yields to broader prenatal populations. In particular, the case mix in a tertiary setting may be enriched for fetuses with more severe renal phenotypes, extrarenal anomalies, or families seeking advanced genetic testing, potentially inflating the observed yield compared with community-based cohorts. Second, although trio-WES substantially improved diagnostic yield in selected subgroups, a proportion of cases with evolving postnatal phenotypes remained genetically unresolved even after reanalysis, reflecting both the intrinsic blind spots of exome-based testing and current constraints in variant interpretation and gene-disease evidence. Third, functional validation was not performed in this study, which may limit confirmation of variant pathogenicity and inference of disease mechanisms, particularly for novel variants or VUS. However, functional studies are often more challenging to implement in the prenatal setting due to limited access to disease-relevant tissue samples and the narrow time window for clinical decision-making.

## 5. Conclusions

In conclusion, our study demonstrates that renal agenesis exhibits marked genetic heterogeneity and that the diagnostic yield of prenatal genetic testing is highly dependent on clinical context. CMA remains an essential first-tier test, whereas trio-WES provides significant incremental value in fetuses with bilateral involvement, non-isolated phenotypes, or extrarenal anomalies. Isolated RA, although associated with a lower diagnostic yield, may still harbor monogenic etiologies and should not be considered genetically benign. An integrated, phenotype-driven diagnostic strategy combined with longitudinal follow-up and data reanalysis offers a rational framework for improving prenatal counseling, refining prognostic assessment, and guiding clinical decision-making in families affected by fetal renal agenesis.

## Figures and Tables

**Table 1:** Clinical characteristics of fetuses with renal agenesis and clinically significant chromosomal abnormalities.

Case	MA (y)	PA (y)	GW (wk)	Sex	Ultrasound Findings	CMA Findings (GRCh37/hg19)	Size (Mb)	Classify	De Novo/Inheritance	Outcomes
1	32	36	26.0	M	RA (L)	arr(X) × 2, (Y) × 1	155.07	P	ND	TOP
2	30	29	26.9	M	RA (L), DK (R), SUA, CPC, ACC, CoA	arr(18) × 3	77.88	P	De novo ^†^	TOP
3	28	28	28.3	F	RA (R)	arr(X) × 3	155.06	P	ND	Live birth
4	28	34	19.0	F	RA (L)	arr(X) × 2~3	155.03	P	Maternal ^†^	LFU
5	28	28	22.0	M	RA (L)	arr22q11.21(20,729,389–21,800,471) × 1	1.07	P	ND	TOP
6	30	31	28.4	F	RA (L)	arr17p12(14,060,335–15,512,120) × 1 *	1.45	P	Paternal ^†^	TOP
7	28	28	24.0	F	RA (R)	arr16p12.2p12.2(21,945,001–22,458,000) × 1 *	0.51	LP	ND	LFU
8	30	32	29.4	M	RA (L)	arr16p11.2(29,510,623–30,243,173) × 1	0.73	P	De novo ^†^	LFU
9	27	28	33.6	M	RA (R), pyelectasis (L)	arr2p25.3p24.2(12,770–18,420,872) × 3	18.41	P	De novo ^†^	TOP
10	28	33	24.0	M	RA (R), RAA	arr2q21.2q32.1(135,000,674–187,740,865) × 3	52.74	P	ND	TOP
11	29	28	29.3	M	RA (R), VM, VSD	arr4q13.1q22.1(61,451,748–93,289,874) × 1	31.84	P	De novo ^†^	TOP
12	27	26	25.1	F	RA (B), oligohydramnios, aortic arch anomaly, pulmonary hypoplasia	arr10q23.33q24.32(96,066,675–103,742,345) × 1	7.68	P	ND	TOP
13	38	39	25.0	F	RA (L), FGR	arr7q11.23(72,717,310–74,565,114) × 1	1.85	P	De novo ^†^	TOP
14	35	38	21.1	M	RA (L), ectopic kidney (R), hemivertebra	arr16p13.11p12.3(15,441,001–18,546,000) × 1 *	3.10	P	Maternal ^†^	TOP
15	35	34	24.0	F	RA (R), FGR	arr10p15.3p14(100,048–11,364,832) × 1	11.26	P	De novo ^†^	TOP
16	26	27	30.0	M	RA (L)	arr8q21.11q22.1(77,270,727–94,187,687) × 2 hmz	16.92	VUS	ND	Live birth
17	35	35	23.6	M	RA (R), SUA, aortic hypoplasia	arr1q42.2q42.3(234,517,914–235,829,843) × 1	1.31	VUS	Maternal ^†^	TOP ^#^
18	25	25	24.1	F	RA (L), hemivertebra	arr9q22.1q22.2(91,438,335–91,981,482) × 1	0.54	VUS	Paternal ^†^	TOP
19	29	32	24.0	F	RA (L), renal dysplasia (R), FGR, oligohydramnios	arr7q35(146,942,939–147,609,292) × 1	0.67	VUS	Maternal ^†^	TOP ^#^

*: Copy number variant detected by CNV-seq; ^†^: variant origin (de novo or inherited) confirmed by trio-WES or parental CMA; ^#^: TOP due to abnormal WES findings; MA: maternal age; PA: paternal age; GW: gestational weeks; CMA: chromosomal microarray analysis; M: male; RA: renal agenesis; L: left; P: pathogenic; ND: not detected; TOP: termination of pregnancy; DK: duplex kidney; R: right; SUA: Single umbilical artery; CPC: choroid plexus cyst; ACC: agenesis of the corpus callosum; CoA: coarctation of aorta; F: female; LFU: lost to follow-up; LP: likely pathogenic; RAA: right aortic arch; VM: ventriculomegaly; VSD: ventricular septal Defect; B: bilateral; FGR: fetal growth restriction; VUS: variants of unknown significance.

**Table 2:** Prenatal genetic profiling of RA cases: WES-based variant spectrum and clinical correlations.

Case	MA (y)	PA (y)	GW (wk)	Sex	Ultrasound Findings	Gene (OMIM)	Reference Sequence	Chromosomal Locus (GRCh37/hg19)	Variant	De Novo or Inheritance	Zygosity	Classify	Disorder	Outcomes
19	29	32	24.0	F	RA (L), renal dysplasia (R), FGR, oligohydramnios	*EYA1* (601653)	NM_000503.6	8:72211435	c.673C>T(p.Gln225Ter)	Paternal	Het	P (PVS1, PM2_Supporting, PP1)	OFC1, BOS1, BOR1	TOP
20	23	31	22.3	M	RA (R)	*KMT2A* (159555)	NM_001197104.2	11:118375905	c.9298G>T(p.Gly3100Ter)	De novo	Het	P (PVS1, PM2_Supporting, PS2_ Supporting)	WDSTS	TOP
21	29	32	20.0	F	RA (B), anhydramnios	*FRAS1* (607830)	NM_025074.7	4:79334189 4:79285209	c.4375C>T(p.Gln1459Ter) c.2722+1G>A	Paternal Maternal	Het Het	LP (PVS1, PM2_Supporting) LP (PVS1_Strong, PM2_Supporting, PM3_Strong)	FRASRS1	TOP
22	28	29	22.0	M	RA (B), anhydramnios, pulmonary hypoplasia, ascites	*FRAS1* (607830)	NM_025074.7	4:79442733 4:79301151	c.10597C>T(p.Arg3533Ter) c.3563+1G>A	Paternal Maternal	Het Het	P (PVS1, PM2_Supporting, PM3) P (PVS1, PM2_Supporting, PM3)	FRASRS1	TOP
23	25	28	17.0	F	RA (R), VM, clubfoot	*FGFR2* (176943)	NM_000141.5	10:123276898	c.1019A>G(p.Tyr340Cys)	De novo	Het	P (PS2, PM5, PP2, PM2_Supporting, PP3_Moderate, PM1_Supporting)	ABS2, ACS I, BSTVS, BBDS1	TOP
24	30	33	27.4	F	RA (R), renal dysplasia (L), oligohydramnios, pulmonary hypoplasia	*PBX1* (176310)	NM_002585.4	1:164781360	c.973_974del(p.Ser325AlafsTer14)	De novo	Het	P (PVS1, PM2_Supporting, PS2_Supporting)	CAKUTHED	TOP
25	32	33	24.4	F	RA (L), TOF, PLSVC, DRP, hypoplastic nasal bone	*PUF60* (604819)	NM_078480.3	8:144900363	c.599_602dup(p.Lys201AsnfsTer21)	De novo	Het	P (PVS1, PS2, PM2_Supporting)	VRJS	TOP
26	28	35	25.4	F	RA (B), oligohydramnios, ARSA, pulmonary hypoplasia, pericardial effusion	*KMT2D* (602113)	NM_003482.3	12:49427729	c.10756_10758del(p.Lys3586del)	De novo	Het	LP (PS2, PM4, PM2_Supporting)	KABUK1, BCAHH	TOP
27	30	29	18.0	F	RA (B), anhydramnios, tricuspid regurgitation, pulmonary artery stenosis, pericardial effusion, FGR, microcephaly	*FRAS1* (607830)	NM_025074.7	4:79186194 4:78987170	c.619C>T(p.Arg207Ter) c.108G>A(p.Ala36=)	Maternal Paternal	Het Het	LP (PVS1, PM2_Supporting) VUS (PM3, PP3, PP4, PM2_Supporting)	FRASRS1	TOP
28	37	38	23.3	F	RA (R), ectopic kidney (L), VM, VSD, PLSVC	*FANCI* (611360)	NM_001113378.2	15:89848570 15:89847103	c.3187-2A>G c.3015G>C(p.Gln1005His)	Maternal Paternal	Het Het	LP (PVS1, PM2_Supporting) VUS (PP3, PM3, PM2_Supporting, PM1_Supporting)	FANCI	TOP
29	30	38	23.4	F	RA (R), FGR, EIF	*LTBP3* (602090)	NM_001130144.3	11:65321357 11:65551414	c.678dup(p.Val227ArgfsTer20) c.1609C>T(p.Arg537Trp)	Paternal Maternal	Het Het	LP (PVS1, PM2_Supporting) VUS (PM2_Supporting, PM3_Supporting)	DASS, GPHYSD3	TOP
17	35	35	23.6	M	RA (R), SUA, aortic hypoplasia	*TBX2* (600747)	NM_005994.4	17:61405528	c.1379del(p.Leu460ArgfsTer208)	De novo	Het	VUS (PS2_Moderate, PM2_Supporting)	VETD	TOP
30	32	31	23.4	M	RA (B), oligohydramnios	*GREB1L* (617782)	NM_001142966.3	18:19070038	c.2756T>C(p.Leu919Pro)	Paternal	Het	VUS (PM2_Supporting, PP3)	DFNA80, RHDA3	TOP
31	28	32	20.7	M	RA (B), oligohydramnios	*OTC* (300461)	NM_000531.6	X:38271187	c.940G>A(p.Glu314Lys)	Maternal	Hemi	VUS (PM2_Supporting, PP3, PM1_Supporting)	OTD	TOP
32	28	25	23.0	M	RA (R)	*RET* (164761)	NM_020975.6	10:43619120	c.2803T>C(p.Trp935Arg)	De novo	Het	VUS (PM2_Supporting, PM1_Supporting, PS2_Supporting, PP3_Moderate)	HSCR1, MTC, MEN2A, MEN2B	TOP
33	33	34	29.6	F	RA (L)	*POLRMT* (601778)	NM_005035.4	19:625123	c.953+1G>C	De novo	Het	VUS (PM2_Supporting, PS2_Supporting)	COXPD55	LFU
34	31	36	31.6	F	RA (L)	*SMC3* (606062)	NM_005445.4	10:112361419	c.2671_2672del(p.Glu891SerfsTer4)	Maternal	Het	VUS (PVS1_Strong, PM2_Supporting)	CDLS3	Live birth
35	40	40	24.0	F	RA (R), ACC	*SCN2A* (182390)	NM_001040142.2	2:166187956	c.2266G>A(p.Val756Ile)	Paternal	Het	VUS (PM2_Supporting, PM1_Supporting, PP3)	DEE11, EA9, BFIS3	TOP
36	25	30	26.4	F	RA (L), FGR, VSD	*PEX7* (601757)	NM_000288.4	6:137143924	c.121G>C(p.Gly41Arg)	Maternal Paternal	Hom	VUS (PP3, PM2_Supporting)	PBD9B, RCDP1	TOP
37	28	28	26.0	F	RA (R)	*PKP2* (602861) *	NM_001005242.3	12:32868926	c.1170+1G>A	Paternal	Het	P (PVS1, PS1, PS4_Supporting)	ARVD9	LFU
38	29	31	24.4	M	RA (L)	*GJB2* (121011) *	NM_004004.6	13:20763612	c.109G>A(p.Val37Ile)	Maternal Paternal	Hom	P (PS4, PP1_Strong, PM3_VeryStrong, PS3_Moderate)	BAPS, DFNA3A, DFNB1A, KIDAD, VOWNKL	Live birth
39	25	27	18.1	M	RA (L)	*GJB2* (121011) *	NM_004004.6	13:20763612	c.109G>A (p.Val37Ile)	Maternal Paternal	Hom	P (PS4, PP1_Strong, PM3_VeryStrong, PS3_Moderate)	BAPS, DFNA3A, DFNB1A, KIDAD, VOWNKL	Live birth

*: Incidental finding; RA: Renal agenesis; MA: Maternal age; PA: Paternal age; GW: Gestational weeks; OFC1: Otofaciocervical syndrome; BOS1: Branchiootic syndrome 1; BOR1: Branchiootorenal syndrome 1, with or without cataracts; F: Female; L: Left; R: Right; FGR: Fetal growth restriction; Het: Heterozygous; P: Pathogenic; TOP: Termination of pregnancy; M: Male; WDSTS: Wiedemann-Steiner syndrome; B: Bilateral; LP: Likely pathogenic; FRASRS1: Fraser syndrome 1; VM: Ventriculomegaly; ABS2: Antley-Bixler syndrome without genital anomalies or disordered steroidogenesis; ACS I: Apert syndrome; BSTVS: Beare-Stevenson cutis gyrata syndrome; BBDS1: Bent bone dysplasia syndrome; CAKUTHED: Congenital anomalies of kidney and urinary tract syndrome with or without hearing loss, abnormal ears, or developmental delay; TOF: Tetralogy of Fallot; PLSVC: Persistent left superior vena cava, DRP: Duplication of renal pelvis; VRJS: Verheij syndrome; ARSA: Aberrant right subclavian artery; KABUK1: Kabuki syndrome 1; BCAHH: Branchial arch abnormalities, choanal atresia, athelia, hearing loss, and hypothyroidism syndrome; VUS: Variants of uncertain significance; VSD: Ventricular septal Defect; FANCI: Fanconi anemia, complementation group I; EIF: Echogenic intracardiac focus; DASS: Dental anomalies and short stature; GPHYSD3: Geleophysic dysplasia 3; SUA: Single umbilical artery; VETD: Vertebral anomalies and variable endocrine and T-cell dysfunction; DFNA80: Deafness, autosomal dominant 80; RHDA3: Renal hypodysplasia/aplasia 3; Hemi: Hemizygous; OTD: Ornithine transcarbamylase deficiency; HSCR1: Hirschsprung disease, susceptibility to 1; MTC: Medullary thyroid carcinoma; MEN2A: Multiple endocrine neoplasia IIA; MEN2B: Multiple endocrine neoplasia IIB; COXPD55: Combined oxidative phosphorylation deficiency 55; LFU: Lost to follow-up; CDLS3: Cornelia de Lange syndrome 3; ACC: Agenesis of the corpus callosum; DEE11: Developmental and epileptic encephalopathy 11; EA9: Episodic ataxia, type 9; BFIS3: Seizures, benign familial infantile, 3; Hom: Homozygous; PBD9B: Peroxisome biogenesis disorder 9B; RCDP1: Rhizomelic chondrodysplasia punctata, type 1; ARVD9: Arrhythmogenic right ventricular dysplasia 9; BAPS: Bart-Pumphrey syndrome; DFNA3A: Deafness, autosomal dominant 3A; DFNB1A: Deafness, autosomal recessive 1A; KIDAD: Keratitis-ichthyosis-deafness syndrome; VOWNKL: Vohwinkel syndrome.

**Table 3:** Summary of isolated renal agenesis cases reported in the present and previous studies.

Case	PMID	First Author	Year	Technique	GW (wk)	Laterality	Variant	Inheritance	Classify	Pregnancy Outcome
1	Our study	Zhang N	2026	CMA	26.0	Left	arr(X) × 2,(Y) × 1	ND	P	TOP
2	Our study	Zhang N	2026	CMA	28.3	Right	arr(X) × 3	ND	P	Live birth
3	Our study	Zhang N	2026	CMA	19.0	Left	arr(X) × 2–3	Mat	P	LFU
4	Our study	Zhang N	2026	CMA	22.0	Left	arr22q11.21(20,729,389–21,800,471) × 1	ND	P	TOP
5	Our study	Zhang N	2026	CNV-seq	28.4	Left	arr17p12(14,060,335–15,512,120) × 1	Pat	P	TOP
6	Our study	Zhang N	2026	CNV-seq	24.0	Right	arr16p12.2(21,945,001–22,458,000) × 1	ND	LP	LFU
7	Our study	Zhang N	2026	CMA	29.4	Left	arr16p11.2(29,510,623–30,243,173) × 1	De novo	P	LFU
8	Our study	Zhang N	2026	WES	22.3	Right	KMT2A (NM_001197104.2): c.9298G>T (p.Gly3100Ter)	De novo	P	TOP
9	Our study	Zhang N	2026	WES	20.0	Bilateral	FRAS1 (NM_025074.7): c.4375C>T (p.Gln1459Ter), c.2722+1G>A	Pat + Mat	LP + LP	TOP
10	40804693	Cai M [12]	2025	CMA	NA	Right	arr16p11.2(28,786,704–29,032,280) × 3	ND	P	TOP
11	40804693	Cai M [12]	2025	CMA	NA	Left	arr17p12(14,083,054–15,482,833) × 1	ND	P	LFU
12	41230579	Du X [11]	2025	CMA	23.1	Right	arr17p12(14,060,293–15,504,668) × 1	ND	P	Liveborn
13	41230579	Du X [11]	2025	CMA	25.6	Right	arr16p13.12–p13.11(14,780,641–16,495,076) × 1	De novo	P	TOP
14	41230579	Du X [11]	2025	CMA	24.9	Right	arr10p15.1–p14(6,533,877–9,022,264) × 1	ND	P	TOP
15	39363241	Li K [9]	2024	CMA	18.0	NA	arr16p11.2(29,628,020–30,332,203) × 1	ND	P	TOP
16	39363241	Li K [9]	2024	CMA	20.0	NA	arr16p11.2(29,656,093–30,328,317) × 1	ND	P	Liveborn
17	39363241	Li K [9]	2024	CMA	23.0	Right	arr22q11.21(18,844,632–21,460,220) × 1	ND	P	TOP
18	34090309	Su J [5]	2022	CMA	25.4	Unilateral	arr22q11.21(21,050,552–21,462,353) × 1	ND	P	TOP
19	34090309	Su J [5]	2022	CMA	24.7	Unilateral	arr22q11.21(18,844,632–21,460,220) × 1	ND	P	TOP
20	34090309	Su J [5]	2022	CMA	26.1	Unilateral	arr16p11.2(29,656,093–30,328,317) × 1	ND	P	Liveborn
21	34090309	Su J [5]	2022	CMA	24.1	Unilateral	arr16p11.2(29,634,212–30,192,561) × 1	ND	P	TOP
22	34090309	Su J [5]	2022	CMA	29.1	Unilateral	arrXp21.1(31,873,913–31,901,450) × 0	ND	P	Liveborn
23	34090309	Su J [5]	2022	CMA	NA	Bilateral	arr10p14(7,768,221–8,972,018) × 1	ND	P	LFU
24	34090309	Su J [5]	2022	CMA	25.3	Unilateral	arr10q11.22q11.23(46,947,635–51,588,177) × 1	ND	P	Liveborn
25	34090309	Su J [5]	2022	CMA	23.7	Unilateral	arr16p13.11(15,052,746–16,303,388) × 1	ND	P	Liveborn
26	33824538	Štefeková A [15]	2022	CMA	22.0	Bilateral	arr22q11.2(21,049,799–21,465,659) × 1	ND	P	TOP
27	29320483	Sagi-Dain L [6]	2018	CMA	NA	Unilateral	arr16p12.2–p11.2(21,894,285–29,315,985) × 1	ND	P	Unknown
28	29320483	Sagi-Dain L [6]	2018	CMA	NA	Unilateral	arr22q11.21(18,636,749–21,800,471) × 1	Mat	P	Unknown
29	35005812	Jordan P [10]	2022	Panel	NA	Bilateral	EYA1 (NM_172060.3): c.1397_1400del (p.Leu466*)	De novo	P	TOP
30	33020172	Arora V [13]	2021	WGS	NA	Bilateral	GFRA1 (NM_005264.5): c.676C>T (p.Arg226*)	Pat + Mat	P	ND
31	33020172	Arora V [13]	2021	WGS	20.0	Bilateral	GFRA1 (NM_005264.5): c.1294delA (p.Thr432Profs*13)	Pat + Mat	P	TOP
32	40223730	Merz LM [8]	2025	WES	ND	Left	HOXC4 (NM_153633.3): c.64_66delGAA (p.Glu22del)	De novo	LP	Unknown
33	40223730	Merz LM [8]	2025	WES	NA	Right	PRPF8 (NM_006445.4): c.4241G>A (p.Arg1414His)	De novo	LP	Unknown
34	40515469	Yao W [28]	2025	WES	15.3	Bilateral	PAX2 (NM_000278.5): c.792G>A (p.Gln264=)	De novo	P	TOP
35	40605465	Feng X [37]	2025	WES	21.6	Bilateral	FREM1 (NM_001379081.2): c.5622G>A (p.Trp1874*), c.3274+4A>G	Pat + Mat	LP + VUS	TOP
36	38410081	Xie F [20]	2024	WES	NA	Bilateral	GREB1L (NM_001142966.3): c.3635del (p.Pro1212Hisfs*21)	De novo	LP	TOP
37	38309594	Fan L [22]	2024	WES	16.0	Bilateral	GREB1L (NM_001142966.3): c.2621G>A (p.Trp874Ter)	mat	LP	TOP
38	34737117	Al-Shamsi B [25]	2022	WES	NA	Bilateral	GFRA1 (NM_005264.8): c.628G>T (p.Gly210Ter)	Pat + Mat	P	ND
39	32643034	Al-Hamed MH [14]	2021	WES	17.0	Bilateral	FREM2 (NM_207361.6): c.2303C>G (p.Ser768*)	Pat + Mat	P	TOP
40	32643034	Al-Hamed MH [14]	2021	WES	NA	Bilateral	FREM2 (NM_207361.6): c.3969delC (p.Asn1323Lysfs*5)	Pat + Mat	P	IUFD
41	32643034	Al-Hamed MH [14]	2021	WES	NA	Bilateral	FRAS1 (NM_025074.7): c.8981dupT (p.His2995Profs*3)	Pat + Mat	P	IUFD
42	32643034	Al-Hamed MH [14]	2021	WES	NA	Bilateral	FRAS1 (NM_025074.7): c.8098+2T>A	Pat + Mat	P	IUFD
43	34049960	Dai L [24]	2021	WES	21.0	Bilateral	NPNT (NM_001184690): exon8:c.777dup (p.Lys260*)	Pat + Mat	P	TOP
44	32598191	Wang A [21]	2020	WES	24.0	Bilateral	GREB1L (NM_001142966.3): c.2333T>A (p.Val778Asp)	Pat	LP	TOP

GW: gestational weeks; CMA: chromosomal microarray analysis; ND: not detected; P: pathogenic; TOP: termination of pregnancy; Mat: Maternal; LFU: lost to follow-up; WES: whole exome sequencing; LP: Likely pathogenic; NA: not available; Pat: Paternal; VUS: Variants of unknown significance; IUFD: Intrauterine fetal death.

## Data Availability

The data that support the findings of this study are not publicly available as the information contained could compromise the privacy of research participants. Further inquiries can be directed to the corresponding author.

## Supplementary Materials

The following supporting information can be downloaded at https://www.mdpi.com/article/10.3390/genes17020176/s1, Supplementary File S1: Analysis and interpretation process of WES data [44,45,46,47,48,49,50,51,52,53,54,55,56,57,58,59,60,61,62,63,64,65,66,67,68,69,70,71,72,73,74,75,76]. Supplementary File S2: Information on all cases included in the literature review.

## Abbreviations

The following abbreviations are used in this manuscript:

RA	Renal Agenesis
CAKUT	Congenital Anomalies of the Kidney and Urinary Tract
URA	Unilateral Renal Agenesis
BRA	Bilateral Renal Agenesis
CNVs	Copy Number Variants
CMA	Chromosomal Microarray Analysis
WES	Whole Exome Sequencing
NGS	Next-Generation Sequencing
GWCMC	Guangzhou Women and Children’s Medical Center
SAPCBD	Specialist Alliance for Prevention and Control of Birth Defects
QF-PCR	Quantitative Fluorescent Polymerase Chain Reaction
CNV-Seq	CNV Sequencing
SNP	Single-Nucleotide Polymorphism
aCGH	Array-based Comparative Genomic Hybridization
VUS	Variants of Uncertain Significance
ACMG	American College of Medical Genetics and Genomics
AMP	Association for Molecular Pathology
P	Pathogenic
LP	Likely Pathogenic
SD	Standard Deviation
SNVs	Single Nucleotide Variants
IFs	Incidental Findings
MRI	Magnetic Resonance Imaging
