# Supplementary material for: Integrated Prenatal Genetic Evaluation of Renal Agenesis: Chromosomal Microarray Analysis, Whole Exome Sequencing, and Outcome Correlations in 203 Fetuses
Source: Genes (Basel). 2026 Jan 31;17(2):176. doi: 10.3390/genes17020176 (PMC12940442; doi:10.3390/genes17020176)
Supplement: Supplementary file 1 [file genes-17-00176-s001.zip › Supplementary File S1.pdf]

Supplementary File S1

Trio-WES was performed in selected cases based on predefined clinical indications, including CNV analysis results that were negative or classified as variants of uncertain significance (VUS), sufficient DNA quantity and quality, and availability of parental samples. In addition, in some cases with advanced gestational age or strong parental preference, trio-WES was performed concurrently with CNV analysis as part of a one-step prenatal diagnostic approach. After informed consent, genomic DNA was extracted from chorionic villi, amniocytes, and cord blood using the Qiagen DNA Blood Midi/Mini kit (Qiagen GmbH, Hilden, Germany) according to the manufacturer's protocol. Blood samples from the parents were obtained concurrently. Targeted enrichment of the DNA sample was conducted using Agilent SureSelect human exome capture probes (V6, Life Technologies, Carlsbad, CA, USA) according to the manufacturer's protocol. The DNA library was sequenced on a HiSeq XTen or Illumina Novaseq 6000 system (Illumina, Inc.) to obtain 150 bp paired-end reads.

Raw fastq reads were filtered by using Trimmomatic (Bolger, Lohse, and Usadel 2014) [44] (v0.36) or fastp (Chen et al. 2018) [45] (v0.20/v0.23) to remove low quality and adapter contaminated reads, leaving clean reads aligned to the human reference genome (GRCh37/Hg19) with BWA (Li and Durbin 2009) [46] (v0.7.17) mem algorithm, with Samtools (Li et al. 2009) [47] (v1.3.1/v1.9) and Picard (v2.17.1) converted to BAM format and PCR duplicates were discarded. Genome Analysis ToolKit (McKenna et al. 2010) [48] (GATK v3.6/v3.8) was employed for local indel realignment, base quality recalibration and haplotypecaller variant calling. Variant annotation was conducted with Ensembl's Variant Effect Predictor (McLaren et al. 2016) [49] (VEP v85/v104) and Annovar (Wang, Li, and Hakonarson 2010) [50] (v2017Jul17/v2020Apr01). Allele frequency information from 1000 Genome Project (1000G Phase 3 v5a), Genome Aggregation Database (gnomAD r2.1/v2.1.1), Exome Aggregation Consortium (ExAC r0.3.1) and the Exome Sequencing Project (ESP v2) were annotated. Multiple software such as SIFT (Sim et al. 2012) [51] , Polyphen2 (Adzhubei et al. 2010) [52], MutationTaster (Schwarz et al. 2014) [53], MutationAssessor (Reva, Antipin, and Sander 2011) [54], Provean (Choi and Chan 2015) [55], CADD (Kircher et al. 2014) [56] and REVEL (Ioannidis et al. 2016) [57] were applied for protein function prediction and Human Splicing Finder (Desmet et al. 2009) [58], MaxEntScan (Yeo and Burge 2004) [59], NNSplice (Reese et al. 1997) [60], GeneSplicer (Pertea, Lin, and Salzberg 2001) [61] and SpliceAI (Jaganathan et al. 2019) [62] were performed to assess potential impacts on splicing. Gene/variants were additionally annotated according to ClinVar, ClinGen, the professional version of the Human Gene Mutation Database (HGMD professional v2018.2 & v2021.2), previously associated diseases (based on Online Mendelian Inheritance in Man and Orphanet), and known functional domain data (according to UniProtKB and Human Protein Reference Database). Besides, the imprinted gene (Geneimprint and MetaImprint database) and genome segmental duplication region (downloaded from the UCSC genome browser) were added to the VCF file annotation. Two aspects were evaluated for gender determination, average depth of specific genes on chromosome Y and the heterozygous variants percentage on chromosome X. Then KING (Manichaikul et al. 2010) [63] and PLINK (Chang et al. 2015) [64] were used to confirm the family pedigree relationship.

Quality control for each sample included an average depth of > 60X and > 90% targeted region with at least 20X in this study. Variants with poor quality were discarded if meeting one of the following criteria, (1) with a depth (DP) <5X; (2) alternate allele proportion (AAP) <0.25; (3) mapping quality (MQ) <40; (4) genotype quality (GQ) <50. All the annotated variants, excluding low quality ones were subject to downstream analysis with the in-house script. Variants with a minor allele frequency (MAF) >5% were filtered out except for those in HGMD, ClinVar and ClinGen BA1 exception list (Ghosh et al. 2018) [65] (BA1). Next, we mainly focused on genomic regions known or likely associated with the disease. Based on VEP functional consequence, potential protein-altering variants (e.g., missense, start loss, stop gain/loss, frameshift, in-frame insertion/deletion, or canonical splice-site) were retained. To aid data interpretation, major indications for WES for each fetus were extracted

from clinical notes and converted into the standard Human Phenotype Ontology (HPO) terms.

A genotype-driven short rare variant list was prioritized for each trio with the help of local population data (more than 10,000 individuals including both patients and healthy individuals), (1) dominant de novo variants; (2) recessive homozygous variants (no homozygotes in the gnomAD2.1 and internal healthy controls); (3) recessive compound heterozygous variants; (4) De novo X chromosome variants or rare hemizygous variants inherited from mother; (5) known disease-causing alleles (ClinVar 3- or 4- star variants); (6) predicted truncating variants (nonsense, frameshift, canonical splice sites) with extremely low allele frequency (<0.01%). This short gene/variant list was then reviewed for clinical correlation and potentially relevant variants were classified based on the American College of Medical Genetics and Genomics (ACMG) guideline (Richards et al. 2015) [66] and ClinGen VCEP gene-specific criteria (when applicable) (Kelly et al. 2018; Gelb et al. 2018; Shen et al. 2019; Oza et al. 2018; Mester et al. 2018; Abou Tayoun et al. 2018; Lee et al. 2018; Zastrow et al. 2018) [66–74]. In addition, *bona fide* disease-causing variants (unrelated to fetal phenotype) with zygosity consistent with disease mode of inheritance in ACMG SF2.0 and childhood-onset disease genes were categorized as potential secondary or incidental findings.

Next, for unsolved cases without a clear answer to the clinical question, a comprehensive review of all rare variants in genes potentially related to clinical indications for prenatal diagnosis was performed with the aid of HPO matching. A gene was considered associated with the fetal anomalies meeting one of the following conditions, the clinical phenotypes of the disease gene should: (1) match HPO entry of the fetal phenotype; (2) match the superclass based on HPO or clinical synopsis in OMIM database; (3) be reported in previous cases manifesting the same or similar phenotypes of the fetuses.

In both steps, WES results were classified into five tiers: (1) positive diagnostic result: P/LP variants identified in a disease gene that can interpret (partly or fully) the fetal phenotype; (2) inconclusive: VUS identified in a disease gene which can explain (partly or fully) the fetal phenotype; (3) incidental findings (IFs): P/LP variants identified in childhood-onset disease gene, unrelated to fetal phenotype; (4) secondary findings (SFs): P/LP variants identified in genes unrelated to fetal phenotype, according to ACMG recommended list (Miller et al. 2021; Kalia et al. 2017) [75,76]; (5) candidate genes: variants (primarily de novo) predicted to be deleterious and absent in general population, identified in undefined disease genes that have a paralog gene or previously published data to support the association with fetal anomalies, or based on animal model and tissue expression.

The WES report included positive diagnostic and inconclusive results related to primary prenatal indications. Incidental and secondary findings with a childhood-onset disease were also included in the report, based on consensus between laboratory and clinicians. Secondary findings with a late-onset disease were not routinely reported.

Tools:

Trimmomatic: http://www.usadellab.org/cms/?page=trimmomatic
Fastp: https://github.com/OpenGene/fastp
BWA: https://bio-bwa.sourceforge.net/
Samtools: https://samtools.sourceforge.net/
Picard: https://broadinstitute.github.io/picard/
Genome Analysis ToolKit: https://gatk.broadinstitute.org/hc/en-us
Ensembl’s Variant Effect Predictor: https://www.ensembl.org/info/docs/tools/vep/index.html
Annovar: https://annovar.openbioinformatics.org/en/latest/
SIFT: https://sift.bii.a-star.edu.sg/

Polyphen2: http://genetics.bwh.harvard.edu/pph2/
MutationTaster: https://www.mutationtaster.org/
MutationAssessor: http://mutationassessor.org/r3/
Provean: https://www.jcvi.org/research/provean
CADD: https://cadd.gs.washington.edu/
REVEL: https://sites.google.com/site/revelgenomics/
Human Splicing Finder: http://www.umd.be/HSF3/HSF.shtml
MaxEntScan: http://hollywood.mit.edu/burgelab/maxent/Xmaxentscan_scoreseq.html
NNSplice: http://www.fruitfly.org/seq_tools/splice.html
GeneSplicer: https://ccb.jhu.edu/software/genesplicer/
SpliceAI: https://github.com/Illumina/SpliceAI
KING: https://www.kingrelatedness.com/manual.shtml
PLINK: https://www.cog-genomics.org/plink/

References

44. Bolger, A.M.; Lohse, M.; Usadel, B. Trimmomatic: a flexible trimmer for Illumina sequence data. *Bioinformatics* **2014**, *30*, 2114–2120. https://doi.org/10.1093/bioinformatics/btu170.
45. Chen, S.; Zhou, Y.; Chen, Y.; Gu, J. fastp: an ultra-fast all-in-one FASTQ preprocessor. *Bioinformatics* **2018**, *34*, i884–i890. https://doi.org/10.1093/bioinformatics/bty560.
46. Li, H.; Durbin, R. Fast and accurate short read alignment with Burrows-Wheeler transform. *Bioinformatics* **2009**, *25*, 1754–1760. https://doi.org/10.1093/bioinformatics/btp324.
47. Li, H.; Handsaker, B.; Wysoker, A.; Fennell, T.; Ruan, J.; Homer, N.; Marth, G.; Abecasis, G.; Durbin, R.; Genome Project Data Processing, S. The Sequence Alignment/Map format and SAMtools. *Bioinformatics* **2009**, *25*, 2078–2079. https://doi.org/10.1093/bioinformatics/btp352.
48. McKenna, A.; Hanna, M.; Banks, E.; Sivachenko, A.; Cibulskis, K.; Kernytsky, A.; Garimella, K.; Altshuler, D.; Gabriel, S.; Daly, M.; et al. The Genome Analysis Toolkit: a MapReduce framework for analyzing next-generation DNA sequencing data. *Genome Res.* **2010**, *20*, 1297–1303. https://doi.org/10.1101/gr.107524.110.
49. McLaren, W.; Gil, L.; Hunt, S.E.; Riat, H.S.; Ritchie, G.R.; Thormann, A.; Flicek, P.; Cunningham, F. The Ensembl Variant Effect Predictor. *Genome Biol.* **2016**, *17*, 122. https://doi.org/10.1186/s13059-016-0974-4.
50. Wang, K.; Li, M.; Hakonarson, H. ANNOVAR: functional annotation of genetic variants from high-throughput sequencing data. *Nucleic Acids Res.* **2010**, *38*, e164. https://doi.org/10.1093/nar/gkq603.
51. Sim, N.L.; Kumar, P.; Hu, J.; Henikoff, S.; Schneider, G.; Ng, P.C. SIFT web server: predicting effects of amino acid substitutions on proteins. *Nucleic Acids Res.* **2012**, *40*, W452–W457. https://doi.org/10.1093/nar/gks539.
52. Adzhubei, I.A.; Schmidt, S.; Peshkin, L.; Ramensky, V.E.; Gerasimova, A.; Bork, P.; Kondrashov, A.S.; Sunyaev, S.R. A method and server for predicting damaging missense mutations. *Nat. Methods* **2010**, *7*, 248–249. https://doi.org/10.1038/nmeth0410-248.
53. Schwarz, J.M.; Cooper, D.N.; Schuelke, M.; Seelow, D. MutationTaster2: mutation prediction for the deep-sequencing age. *Nat. Methods* **2014**, *11*, 361–362. https://doi.org/10.1038/nmeth.2890.
54. Reva, B.; Antipin, Y.; Sander, C. Predicting the functional impact of protein mutations: application to cancer genomics. *Nucleic Acids Res.* **2011**, *39*, e118. https://doi.org/10.1093/nar/gkr407.
55. Choi, Y.; Chan, A.P. PROVEAN web server: a tool to predict the functional effect of amino acid substitutions and indels. *Bioinformatics* **2015**, *31*, 2745–2747. https://doi.org/10.1093/bioinformatics/btv195.
56. Kircher, M.; Witten, D.M.; Jain, P.; O'Roak, B.J.; Cooper, G.M.; Shendure, J. A general framework for estimating the relative pathogenicity of human genetic variants. *Nat. Genet.* **2014**, *46*, 310–315. https://doi.org/10.1038/ng.2892.
57. Ioannidis, N.M.; Rothstein, J.H.; Pejaver, V.; Middha, S.; McDonnell, S.K.; Baheti, S.; Musolf, A.; Li, Q.; Holzinger, E.; Karyadi, D.; et al. REVEL: An Ensemble Method for Predicting the Pathogenicity of Rare Missense Variants. *Am. J. Hum. Genet.* **2016**, *99*, 877–885. https://doi.org/10.1016/j.ajhg.2016.08.016.
58. Desmet, F.O.; Hamroun, D.; Lalande, M.; Collod-Beroud, G.; Claustres, M.; Beroud, C. Human Splicing Finder: an online bioinformatics tool to predict splicing signals. *Nucleic Acids Res.* **2009**, *37*, e67. https://doi.org/10.1093/nar/gkp215.
59. Yeo, G.; Burge, C.B. Maximum entropy modeling of short sequence motifs with applications to RNA splicing signals. *J. Comput. Biol.* **2004**, *11*, 377–394. https://doi.org/10.1089/1066527041410418.
60. Reese, M.G.; Eeckman, F.H.; Kulp, D.; Haussler, D. Improved splice site detection in Genie. *J. Comput. Biol.* **1997**, *4*, 311–323. https://doi.org/10.1089/cmb.1997.4.311.
61. Pertea, M.; Lin, X.; Salzberg, S.L. GeneSplicer: a new computational method for splice site prediction. *Nucleic Acids Res.* **2001**, *29*, 1185–1190. https://doi.org/10.1093/nar/29.5.1185.
62. Jaganathan, K.; Kyriazopoulou Panagiotopoulou, S.; McRae, J.F.; Darbandi, S.F.; Knowles, D.; Li, Y.I.; Kosmicki, J.A.; Arbelaez, J.; Cui, W.; Schwartz, G.B.; et al. Predicting Splicing from Primary Sequence with Deep Learning. *Cell* **2019**, *176*, 535–548.e524. https://doi.org/10.1016/j.cell.2018.12.015.
63. Manichaikul, A.; Mychaleckyj, J.C.; Rich, S.S.; Daly, K.; Sale, M.; Chen, W.M. Robust relationship inference in genome-wide association studies. *Bioinformatics* **2010**, *26*, 2867–2873. https://doi.org/10.1093/bioinformatics/btq559.

64. Chang, C.C.; Chow, C.C.; Tellier, L.C.; Vattikuti, S.; Purcell, S.M.; Lee, J.J. Second-generation PLINK: rising to the challenge of larger and richer datasets. *Gigascience* **2015**, *4*, 7. https://doi.org/10.1186/s13742-015-0047-8.
65. Ghosh, R.; Harrison, S.M.; Rehm, H.L.; Plon, S.E.; Biesecker, L.G.; ClinGen Sequence Variant Interpretation Working, G. Updated recommendation for the benign stand-alone ACMG/AMP criterion. *Hum. Mutat.* **2018**, *39*, 1525–1530. https://doi.org/10.1002/humu.23642.
66. Richards, S.; Aziz, N.; Bale, S.; Bick, D.; Das, S.; Gastier-Foster, J.; Grody, W.W.; Hegde, M.; Lyon, E.; Spector, E.; et al. Standards and guidelines for the interpretation of sequence variants: a joint consensus recommendation of the American College of Medical Genetics and Genomics and the Association for Molecular Pathology. *Genet. Med.* **2015**, *17*, 405–424. https://doi.org/10.1038/gim.2015.30.
67. Kelly, M.A.; Caleshu, C.; Morales, A.; Buchan, J.; Wolf, Z.; Harrison, S.M.; Cook, S.; Dillon, M.W.; Garcia, J.; Haverfield, E.; et al. Adaptation and validation of the ACMG/AMP variant classification framework for MYH7-associated inherited cardiomyopathies: recommendations by ClinGen's Inherited Cardiomyopathy *Expert Panel. Genet Med.* **2018**, *20*, 351–359. https://doi.org/10.1038/gim.2017.218.
68. Gelb, B.D.; Cave, H.; Dillon, M.W.; Gripp, K.W.; Lee, J.A.; Mason-Suares, H.; Rauen, K.A.; Williams, B.; Zenker, M.; Vincent, L.M.; et al. ClinGen's RASopathy Expert Panel consensus methods for variant interpretation. *Genet. Med.* **2018**, *20*, 1334–1345. https://doi.org/10.1038/gim.2018.3.
69. Shen, J.; Oza, A.M.; Del Castillo, I.; Duzkale, H.; Matsunaga, T.; Pandya, A.; Kang, H.P.; Mar-Heyming, R.; Guha, S.; Moyer, K.; et al. Consensus interpretation of the p.Met34Thr and p.Val37Ile variants in GJB2 by the ClinGen Hearing Loss Expert Panel. *Genet. Med.* **2019**, *21*, 2442–2452. https://doi.org/10.1038/s41436-019-0535-9.
70. Oza, A.M.; DiStefano, M.T.; Hemphill, S.E.; Cushman, B.J.; Grant, A.R.; Siegert, R.K.; Shen, J.; Chapin, A.; Boczek, N.J.; Schimmenti, L.A.; et al. Expert specification of the ACMG/AMP variant interpretation guidelines for genetic hearing loss. *Hum. Mutat.* **2018**, *39*, 1593–1613. https://doi.org/10.1002/humu.23630.
71. Mester, J.L.; Ghosh, R.; Pesaran, T.; Huether, R.; Karam, R.; Hruska, K.S.; Costa, H.A.; Lachlan, K.; Ngeow, J.; Barnholtz-Sloan, J.; et al. Gene-specific criteria for PTEN variant curation: Recommendations from the ClinGen PTEN Expert Panel. *Hum. Mutat.* **2018**, *39*, 1581–1592. https://doi.org/10.1002/humu.23636.
72. Abou Tayoun, A.N.; Pesaran, T.; DiStefano, M.T.; Oza, A.; Rehm, H.L.; Biesecker, L.G.; Harrison, S.M.; ClinGen Sequence Variant Interpretation Working, G. Recommendations for interpreting the loss of function PVS1 ACMG/AMP variant criterion. *Hum. Mutat.* **2018**, *39*, 1517–1524. https://doi.org/10.1002/humu.23626.
73. Lee, K.; Krempely, K.; Roberts, M.E.; Anderson, M.J.; Carneiro, F.; Chao, E.; Dixon, K.; Figueiredo, J.; Ghosh, R.; Huntsman, D.; et al. Specifications of the ACMG/AMP variant curation guidelines for the analysis of germline CDH1 sequence variants. *Hum. Mutat.* **2018**, *39*, 1553–1568. https://doi.org/10.1002/humu.23650.
74. Zastrow, D.B.; Baudet, H.; Shen, W.; Thomas, A.; Si, Y.; Weaver, M.A.; Lager, A.M.; Liu, J.; Mangels, R.; Dwight, S.S.; et al. Unique aspects of sequence variant interpretation for inborn errors of metabolism (IEM): The ClinGen IEM Working Group and the Phenylalanine Hydroxylase Gene. *Hum. Mutat.* **2018**, *39*, 1569–1580. https://doi.org/10.1002/humu.23649.
75. Miller, D.T.; Lee, K.; Chung, W.K.; Gordon, A.S.; Herman, G.E.; Klein, T.E.; Stewart, D.R.; Amendola, L.M.; Adelman, K.; Bale, S.J.; et al. ACMG SF v3.0 list for reporting of secondary findings in clinical exome and genome sequencing: a policy statement of the American College of Medical Genetics and Genomics (ACMG). *Genet. Med.* **2021**, *23*, 1381–1390. https://doi.org/10.1038/s41436-021-01172-3.
76. Kalia, S.S.; Adelman, K.; Bale, S.J.; Chung, W.K.; Eng, C.; Evans, J.P.; Herman, G.E.; Hufnagel, S.B.; Klein, T.E.; Korf, B.R.; et al. Recommendations for reporting of secondary findings in clinical exome and genome sequencing, 2016 update (ACMG SF v2.0): a policy statement of the American College of Medical Genetics and Genomics. *Genet. Med.* **2017**, *19*, 249–255. https://doi.org/10.1038/gim.2016.190.
77.
